# Climate action for health and wellbeing in cities: a protocol for the systematic development of a database of peer-reviewed studies using machine learning methods

**DOI:** 10.12688/wellcomeopenres.16570.1

**Published:** 2021-03-05

**Authors:** Kristine Belesova, Max Callaghan, Jan C Minx, Felix Creutzig, Catalina Turcu, Emma Hutchinson, James Milner, Melanie Crane, Andy Haines, Michael Davies, Paul Wilkinson

**Affiliations:** 1 Department of Public Health, Environments and Society and Centre on Climate Change and Planetary Health, London School of Hygiene and Tropical Medicine, London, WC1H 9SH, UK; 2 Mercator Research Institute on Global Commons and Climate Change, Berlin, 10829, Germany; 3 Bartlett School of Planning, University College London, London, WC1H 0QB, UK; 4 Charles Perkins Centre, Sydney School of Public Health, University of Sydney, Sydney, Australia; 5 Bartlett School Environment, Energy & Resources, University College London, London, WC1H 0QB, UK

**Keywords:** climate change, cities, urban health, mitigation, adaptation, planetary health, case studies, implementation, wellbeing, public health, climate action, evaluation, intervention, solutions, actions

## Abstract

Cities produce more than 70% of global greenhouse gas emissions. Action by cities is therefore crucial for climate change mitigation as well as for safeguarding the health and wellbeing of their populations under climate change. Many city governments have made ambitious commitments to climate change mitigation and adaptation and implemented a range of actions to address them. However, a systematic record and synthesis of the findings of evaluations of the effect of such actions on human health and wellbeing is currently lacking. This, in turn, impedes the development of robust knowledge on what constitutes high-impact climate actions of benefit to human health and wellbeing, which can inform future action plans, their implementation and scale-up. The development of a systematic record of studies reporting climate and health actions in cities is made challenging by the broad landscape of relevant literature scattered across many disciplines and sectors, which is challenging to effectively consolidate using traditional literature review methods. This protocol reports an innovative approach for the systematic development of a database of studies of climate change mitigation and adaptation actions implemented in cities, and their benefits (or disbenefits) for human health and wellbeing, derived from peer-reviewed academic literature. Our approach draws on extensive tailored search strategies and machine learning methods for article classification and tagging to generate a database for subsequent systematic reviews addressing questions of importance to urban decision-makers on climate actions in cities for human health and wellbeing.

## Introduction

Cities are responsible for 71% to 76% of global energy-related carbon emissions, including both consumption and production-related emission ( Seto *et al.*, 2014). To limit global average temperature increase to well below 2°C, CO _2_ and short-lived climate pollutant emissions need to be reduced to net zero (often abbreviated as the net zero target) within the next 50 years – though some suggest that cities should achieve this much earlier ( C40 & Arup, 2017). The achievement of this deadline would require climate action at all scales: individual, city, national and international levels triggering rapid transformation of the ways in which urban societies operate.

In 2015, there were over 10,000 climate actions identified as being undertaken in the 96 cities comprising the C40 cities climate leadership group, with further potential 26,000 actions identified that could be implemented to expand their range of existing climate actions ( C40 Cities & Arup, 2015). However, so far urban climate actions have not resulted in rapid and sustained emission reductions that are required to meet the climate goals of the Paris Agreement ( Reckien *et al.*, 2014). The overall progress on climate change action so far has been seriously inadequate in comparison to the magnitude of the challenge, as global greenhouse gas emissions have been steadily rising. Although a decline of 4 to 7% (2% to 13%) in global CO2 emissions is projected in 2020 due to the measures taken in response to the COVID-19 pandemic, those are not suitable for the required sustained long-term emission reduction ( Belesova *et al.*, 2020b; Forster *et al.*, 2020; Quéré *et al.*, 2020).

Climate change mitigation and adaptation actions have implications for human health and wellbeing in cities (see Box 1 for definitions). Mitigation actions can produce considerable health and wellbeing benefits for urban residents, for example, through reductions in air pollution, increased levels of active travel, reduced noise levels, more urban greenspace ( Gao *et al.*, 2018). Adaptation actions can benefit health through, for example, reduced risk of extreme weather effects, exposure to vector-borne infectious diseases and greater resilience to socio-economic shocks ( Smith *et al.*, 2014). Nevertheless, certain climate actions and their implementation may also result in unintended adverse consequences e.g., the use of air conditioning to manage thermal comfort during heatwaves leads to increased electricity consumption and further exacerbation of urban heat reducing the overall health benefits of reduced indoor heat exposure; likewise, building retrofit could, if not implemented correctly, lead, for example, to poor ventilation and accumulation of pollutants generated indoors with adverse effects on health ( Li *et al.*, 2014; Shrubsole *et al.*, 2014; Wenz *et al.*, 2017).

**Box 1. Definitions of key concepts**

• **Mitigation**: actions that aim to reduce sources or enhance sinks of greenhouse gases ( IPCC, 2012).

• **Adaptation**: "the process of adjustment to actual or expected climate and its effects" ( IPCC, 2012).

• **Action**: tangible actions to alter institutions, technology, policies, programs, built environments, mandates or behaviours in the effort to reduce the rate of climate change and/or adapt to it ( Lesnikowski *et al.*, 2011; Lesnikowski *et al.*, 2013). *

• **Health**: “health is a state of complete physical, mental and social well-being and not merely the absence of disease or infirmity” ( World Health Organization (WHO), 1948). Although the WHO definition encompasses physical, mental and social health and wellbeing, here we largely reserve the term “health outcomes” for adverse physical and mental health outcomes and use the term ‘wellbeing’ separately.

• **Wellbeing**: here is used to emphasise the wider psychological and social aspects of human health that determine subjective human wellbeing, as defined by Marsh *et al.* (2020), understood as a cognitive sense of life satisfaction and pleasant or unpleasant affect (moods and emotions) ( Dodge *et al.*, 2012).

*As a part of action we also include "groundwork activities", i.e., activities that prepare conditions for mitigation/adaptation, enable mitigation and adaptation actions, inform and prepare stakeholders for the actions, e.g., vulnerability assessments, adaptation and mitigation research, development of conceptual tools, stakeholder networking, and provision of policy recommendations ( Lesnikowski *et al.*, 2011; Lesnikowski *et al.*, 2013). These often contribute to the capacity to mitigate or adapt to climate change.

To our knowledge, there are no comprehensive assessments of collective ‘real world’ climate actions and their impacts on human health and well-being in cities, including comprehensive databases of publications available for syntheses or syntheses themselves. Lamb *et al.* used machine learning methods to develop a database of 4,000 urban climate mitigation case studies reported in peer-reviewed literature ( Lamb *et al.*, 2019), but their approach does not include climate change adaptation terms, nor the implications of climate actions for human health and wellbeing. The lack of comprehensive syntheses addressing stakeholder needs hinders evidence-based decision-making on, and effective implementation of, the most reliable high-impact actions.

The following protocol presents an approach for the systematic development of a database of peer-reviewed studies to enable synthesising evidence on climate actions implemented in cities and their effects on human health and wellbeing. Identifying such studies requires investigation across a wide pool of literature dispersed across many disciplines, sectors, and topics. The capacity of traditional systematic literature review processes to comprehensively capture this extensive literature is limited. Therefore, here, we extend the machine-learning methods developed by Lamb *et al.* (2019), and apply them to a set of extensive specialised search strategies developed by our multi-disciplinary team to cover studies of both urban climate change mitigation and adaptation actions relevant to human health and wellbeing. Machine learning entails training a computer to perform some parts of work automatically ( Berrang-Ford *et al.*, 2021). In the case of systematic literature identification, machine learning can be used to reduce human resources needed to search, screen, and classify tens of thousands of scientific articles, and hence, cover a much broader scope of literature ( Berrang-Ford *et al.*, 2021). This protocol provides an overview of the process and methods for developing the database of studies.

This database is being developed as a part of the Complex Urban Systems for Sustainability and Health (CUSSH) project in collaboration with lead authors of the upcoming 6 ^th^ Assessment Report of the Intergovernmental Panel on Climate Change (IPCC). The CUSSH project is a Wellcome Trust funded five-year consortium of 13 academic and other institutions collaborating with six cities – London, Rennes, Nairobi, Kisumu, Beijing, and Ningbo – the C40 Cities Climate Leadership Network, and other stakeholders. Its aim is to deliver key global research on the systems that connect urban sustainable development and population health ( Belesova *et al.*, 2018). The database will provide evidence to improve understanding of a range of questions about urban mitigation/ adaptation actions and human health impacts — relevant to cities around the world, and to urban policy makers and NGOs who require examples of good practice in climate action, city networks such as C40, and the IPCC. The IPCC increasingly pays attention to demand-side solutions that require evaluation in terms of their impact on health and wellbeing ( Creutzig *et al.*, 2018). As most effects of climate action will take place at the scale of human urban settlements, a comprehensive database of the underlying literature will hence be invaluable.

## Aim and research areas

Our aim is to test and implement an approach for the systematic development of a database of urban climate change mitigation and adaptation actions (see the definition in Box 1) relevant to human health and wellbeing that have been implemented in cities and reported in peer-reviewed academic literature. Subsequently, we intend to use this database to address questions about current climate mitigation and adaptation actions for health and well-being that are implemented in cities and reported in peer-reviewed literature in at least three broad areas:

1. Distribution, variation, and diversity of actions across the world's cities
2. The scale of impact and effectiveness of the actions
3. Lessons for their successful implementation

We particularly seek input from policy makers and other urban stakeholders to ensure the relevance of our research questions and strengthen the translational relevance of the evidence to policy and practice. Protocols for each of the subsequent systematic reviews will be reported separately. This protocol covers only the approach to the development of the source database itself. This protocol has been registered in the OSF Registries ( Belesova *et al.*, 2020a).

## Scope of the database

The scope of the database is framed by the PICO statement and inclusion criteria elaborated below.

### PICO statement

**Population**: populations in any of the cities included in the *GeoNames* database ( GeoNames Team, 2020)

**Intervention**: actions targeting either climate change mitigation and/or adaptation

**Comparison**: any form of comparison, including, but not limited to change-over-time and between-area controlled studies, as well as descriptive reports 

**Outcome**:

(1) any form of documented health and wellbeing outcome, whether positive or negative ^1^,
(2) change in policy ambitions or in the drivers and processes contributing to climate change mitigation and/or adaptation

### Inclusion criteria

Papers will be included according to the following criteria:

- **Literature type:** peer-reviewed scientific papers reporting original research;
- **Year of publication:** from 1990 until present to capture literature produced after the publication in year 1990 of the first assessment report by the IPCC;
- **Language:** English;
- **Study type:** any observational, evaluation, natural experiment or modelling study that reports any action(s) that
  - target **climate change** mitigation and/or adaptation;
  - report actual or potential impact on health and/or wellbeing;
  - are intended to have impact at the level of the whole city or substantial part of the city (neighbourhood-level and larger);
  - have been **implemented**, i.e., their implementation is ongoing, completed, failed.

### Exclusion criteria:

- **Literature type:** non peer-reviewed literature, commentaries, editorials, systematic and non-systematic literature reviews, opinion pieces, news reports, book chapters, meeting reports/conference proceedings, grey literature;
- **Year of publication:** before 1990;
- **Language:** other than English;
- **Study type:** studies that do not report implementation of a relevant action(-s) (as per inclusion criteria above), e.g., association studies of an environmental exposure with an outcome but without an intervention (e.g., studies of the heat—mortality association), methodological studies, carbon accounting studies, conceptual and protocol papers, in silico modelling actions that have not been implemented, i.e., action plans, masterplans, scenarios, scenario modelling, urban visions, guidelines, frameworks (N.B. modelling studies that evaluate impacts of an existing intervention are included, e.g., modelling of the effects of intervention-related changes in air pollution),
- **Scale:** small scale, niche action or pilot studies not intended to have impact at the level of the whole city or substantial part of the city (i.e., actions at scales below neighbourhood-level);
- **Implementation:** actions that are only hypothesized or planned and not yet implemented.

We will include only anticipatory and purposeful adaptation and mitigation and exclude unplanned/ spontaneous efforts (co-)resulting in adaptation and mitigation or entirely hypothetical actions with no attempt at implementation. Hence, the database will focus on tangible actions and preparatory activities specifically intended to reduce GHG emissions or the adverse effects of climate change which report potential or actual impacts on human health and wellbeing.

## Literature search, screening, and data management

**Figure 1.**
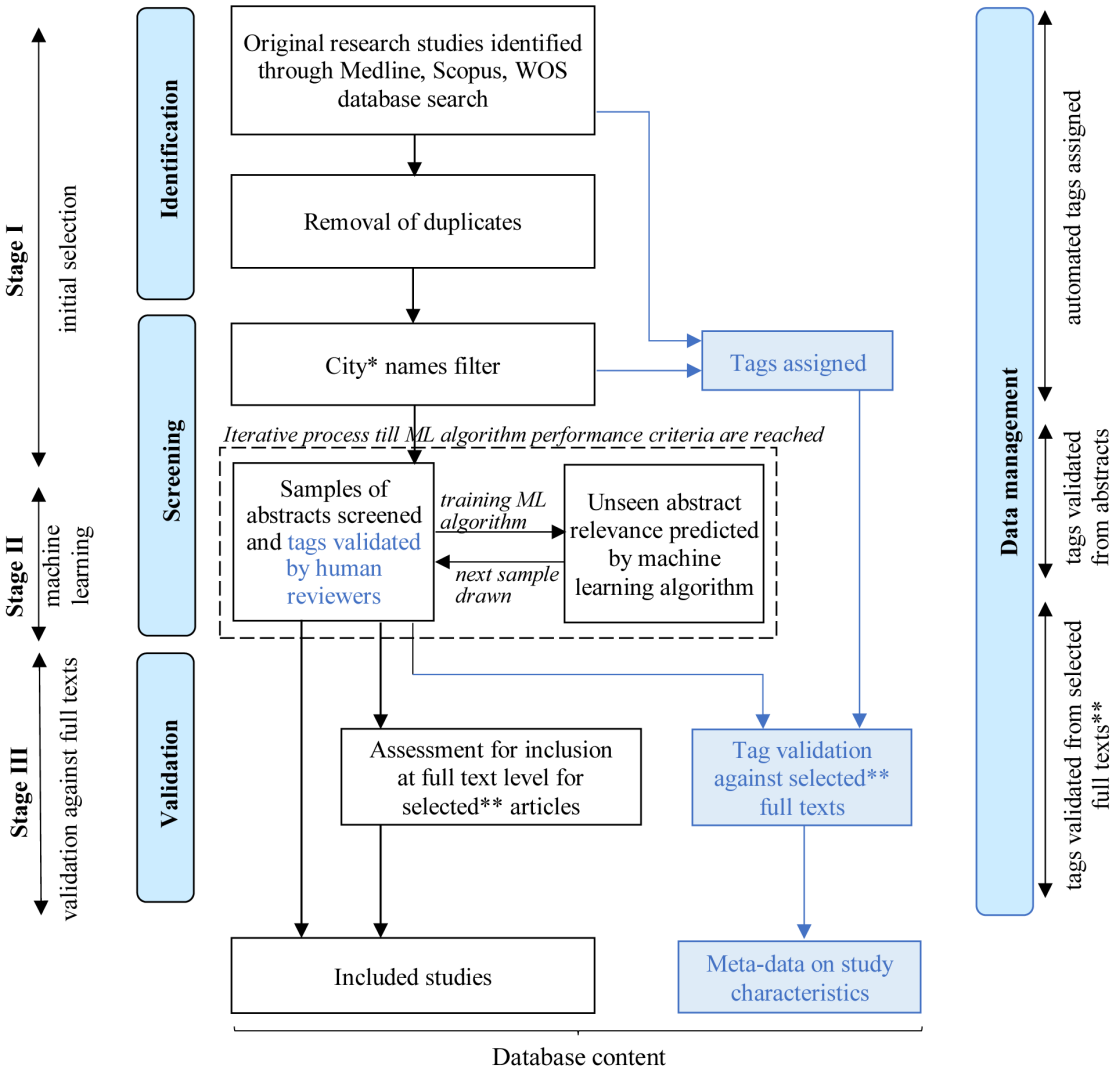
A diagram of the review and database development process. ML: machine learning; WOS: Web of Science. *also includes names of major city networks. **selected articles include the articles whose inclusion was not decided, and a random sample of the articles included following the abstract level assessment.

Our three-stage process of study identification (initial selection, machine learning, and eligibility assessment) will be done in parallel to data management, with both being supported by machine learning and human processing ( Figure 1).

### Stage I: initial selection

We will make use of a search that has been performed across Web of Science Core Collections (consisting of SCI-EXPANDED, SSCI, A&HCI, CPCI-S, CPCI-SSH, and ESCI), Scopus, and Medline (accessed via Web of Science). These databases allow access to literature from diverse disciplinary perspectives, matching the transdisciplinary nature of our research. With the breadth of our search results approximating a total of 650,000 records (including duplicates and before applying the city filter), we do not search an even wider set of databases due to a lack of an automated workflow that would support other bibliographic databases and grey literature search within our current technical infrastructure and capabilities.

**Figure 2.**
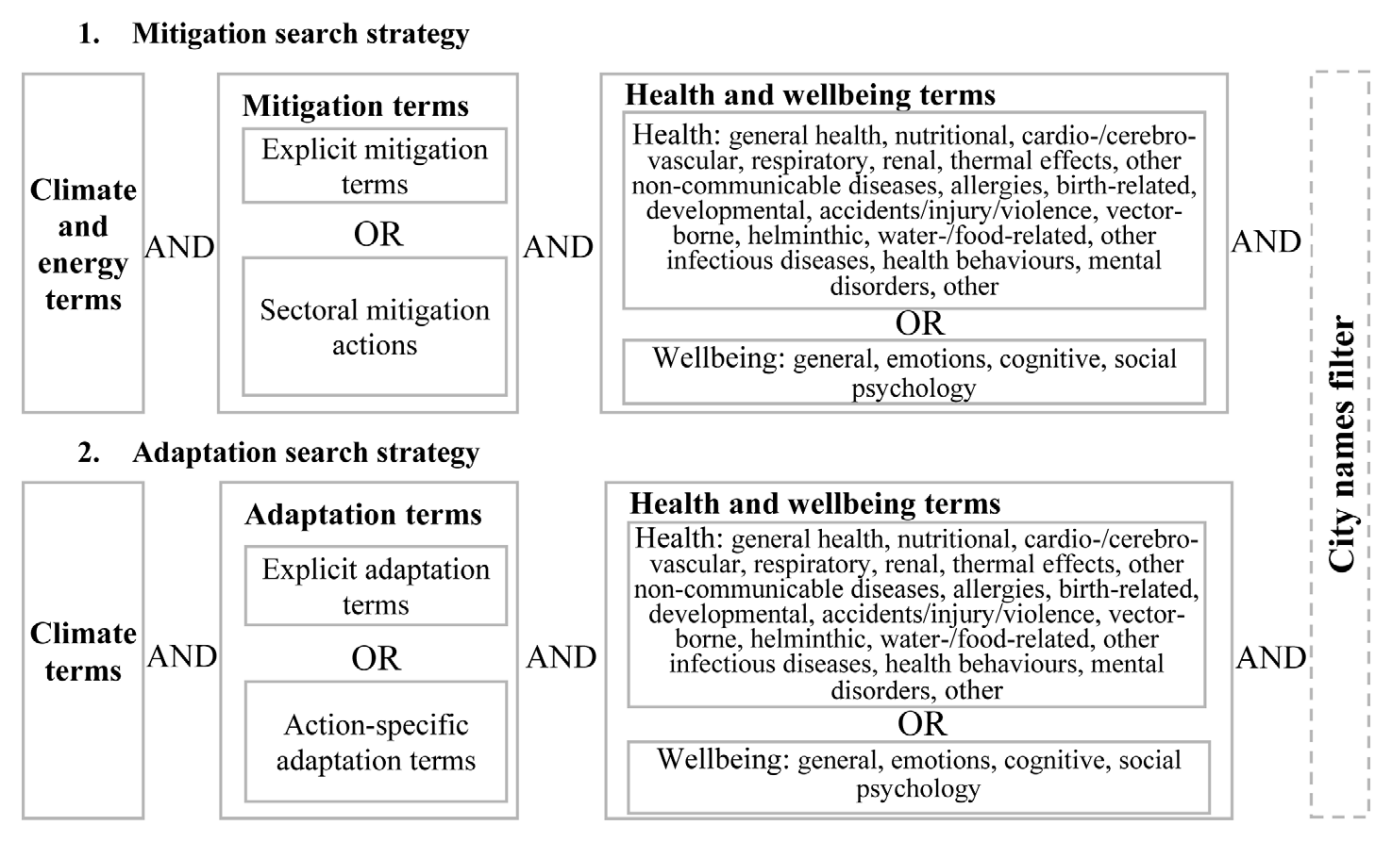
Search strategy structure.

We have developed two separate sets of search terms, one to capture climate change adaptation actions and one climate change mitigation actions ( Figure 2). Papers describing actions that are taken to contribute to climate change mitigation or adaptation may not always mention the terms ‘mitigation’ or ‘adaptation’ or their synonyms (e.g., ‘emission reduction’ and ‘climate risk reduction’) explicitly. Therefore, both search strategies contain terms for actions likely to entail mitigation and/or adaptation effects even if ‘adaptation’ or ‘mitigation’ is not mentioned. Such terms include, for example, ‘energy efficiency policies’ and ‘coastal protection’.

The structure of the search terms is shown in Figure 2. Both searches entail the intersection (‘AND’) of three blocks of terms, representing climate, mitigation/adaptation actions, and health/wellbeing. Specifically, for mitigation the blocks of terms are: (climate terms OR energy terms) AND (explicit mitigation and mitigation policy terms OR sector-specific mitigation terms) AND (health terms OR wellbeing terms). For adaptation the terms are: (climate terms) AND (explicit adaptation terms including resilience OR action-specific adaptation terms) AND (health terms OR wellbeing terms). The sector-specific mitigation terms are grouped by the following ‘sectors’: transport, buildings, urban form and greenspace, waste and food. The action-specific adaptation terms follow sets of adaptation strategies that can be implemented for each category of climate change impacts on human health outlined in the health chapter of the 5 ^th^ assessment report of the IPCC with further sectoral terms of relevance to health ( Smith *et al.*, 2014). Thus, the mitigation search strategy targets all climate change mitigation actions mentioning health or human wellbeing terms in their abstract/title or keywords. The adaptation search strategy targets those actions that have the potential to protect people from health risks of climate change (e.g., coastal defences) and does not cover actions implemented to maintain stability of other sectors (e.g., profits in the tourism sector) without mentioning human health or wellbeing. In the health and wellbeing block of terms, we did not attempt to include terms for well-known exposures through which mitigation and adaptation actions affect human health, e.g., air pollution. Inclusion of only few well-known exposure terms would have biased our search results towards well-known actions and impact mechanisms; inclusion of terms for all possible exposures and impact pathways would have been impractical due to the extensive range of potential pathways. We also did not include terms for long-term health effects, such as cancers, the rates of which can change with the implementation of certain mitigation strategies, as they do not reflect health co-benefits of climate change mitigation – a concept that refers to the benefits occurring the near-term, and thus, providing an additional incentive for the implementation of mitigation actions ( West *et al.*, 2013).

The adaptation terms were constructed by KB with additions and edits from all co-authors. The mitigation terms were constructed by JM, FC and KB with additions and edits from all co-authors. Terms were selected based on keystone articles and authors’ expertise, which covers all major urban sectors (transport, buildings, urban form including green and blue space, food systems, waste, energy, water and sanitation, health care, industry) and relevant disciplines (epidemiology, public health, urban planning, building science, climate change and energy, sustainability science, and implementation science). Climate terms for the mitigation search were adapted from Grieneisen & Zhang (2011) and for the adaptation search from Whitmee *et al.* (2015). Adaptation terms for the food sector were adapted from Aleksandrowicz *et al.* (2016). Health terms were adapted from Berrang-Ford *et al.* (2021) with additions based on reviewing the International Statistical Classification of Diseases and Related Health Problems 10 ^th^ Revision ( World Health Organization (WHO), 2019) for terms of potential relevance. Wellbeing terms were constructed by KB drawing on the determinants of subjective wellbeing ( Marsh *et al.*, 2020) and published search strategies on mental health and wellbeing ( Clark & Paunovic, 2018; Cochrane Common Mental Disorders, 2014; Keynejad *et al.*, 2020; Larvin *et al.*, 2019; Wilczynski *et al.*, 2006).

When constructing the search strategy, we tested the search term relevance and performance of different combinations of search terms, identifying and restricting any terms that contributed excessive numbers of irrelevant results. The search strategy was designed to be comprehensive and allow identifying understudied mitigation and adaptation actions.

Search terms were reviewed by all team members and peer-reviewed by a librarian experienced in systematic review searching, using the PRESS Guidance ( McGowan *et al.*, 2016). Recommendations were discussed with the project team and implemented where appropriate. The final set of search terms developed for Medline is available as extended data ( Belesova *et al.*, 2021). The search strategies were adapted to the syntax of each bibliographic database. The bibliographic databases were searched by article abstracts, titles, key words, and headings in Scopus and Medline and by article abstracts, titles in Web of Science. Keyword searches in Web of Science were excluded as their inbuilt "keyword plus" function generates additional default searches that are not transparent and generate a large number of irrelevant records ( Berrang-Ford *et al.*, 2021).

We will run the searches restricting them to the original research study articles published in English. We will remove duplicates and discard records where the abstract is missing, as the abstracts are essential to decide on study inclusion into the database. To identify actions implemented in urban areas, we will search the selected abstracts for names of cities and city networks. We use the Geonames database of geographic locations, which aggregates national survey data, travel destinations and open-sourced contributions, specifying a global list of cities with populations greater than 15,000. To this list we will add names of such city networks as C40 city leadership group, Multi-City Multi-Country (MCC) collaborative research network, ILCEI – Local Governments for Sustainability, Covenant of Mayors, 100 Resilient Cities, and others.

### Stage II: machine learning assisted screening

We will use supervised machine learning (ML) to accelerate the screening process through an iterative cycle of training, testing against manual classification and re-training of the ML algorithm until a stopping rule is met ( Callaghan & Müller-Hansen, 2019). We will use NACSOS research platform for screening process and Python scikit-learn library for machine learning ( Callaghan *et al*., 2020; Pedregosa *et al.*, 2012).

**Figure 3.**
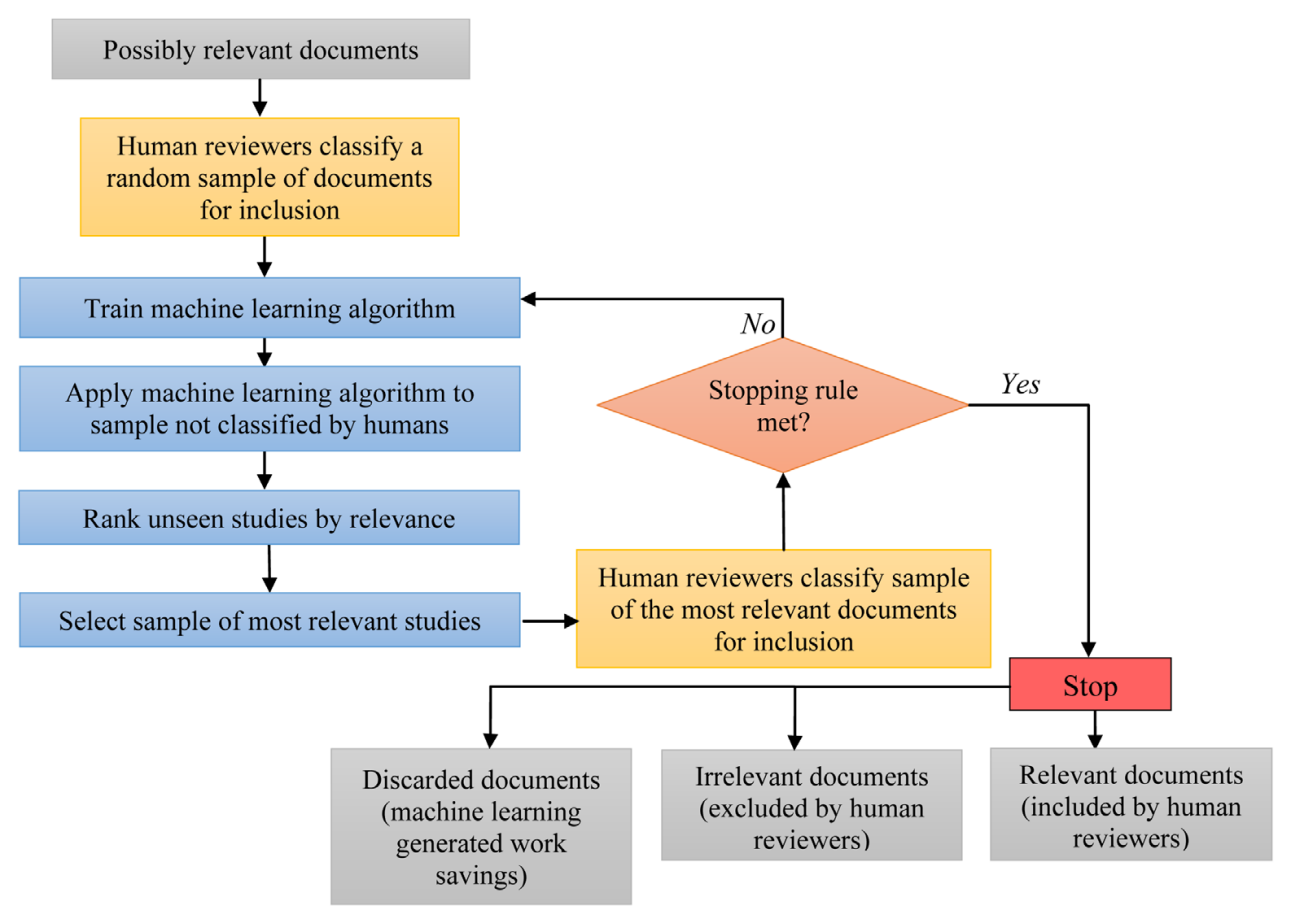
Flowchart illustrating the process of the machine learning assisted abstract screening ( Callaghan & Müller-Hansen, 2019).

The process is illustrated in Figure 3. First, a random sample of the studies selected by the initial literature search is screened by two human reviewers and classified with respect to relevance, with any inconsistencies between reviewers resolved by a third reviewer as necessary. This classification is treated as the ‘gold standard’ classification of relevance (‘the truth’). The classified random sample is then used to train a machine learning algorithm, which, once trained, is applied to predict the relevance of the remaining studies unseen by human reviewers, assigning to each study a score from 0 to 1 for the likelihood of relevance.

The studies with the highest ML scores (those with a score closest to 1) are then reviewed by two independent human reviewers whose classification is used to define the proportion of the high ML-score sample that was correctly classified as truly relevant.

The human (‘gold standard’) classification of this sample is then used to improve the training of the ML algorithm in an iterative process of further ML training, re-classification of studies and testing the results against by human reviewers.

After each cycle, a test is applied of whether to continue the iterative cycles or stop. The stopping rule is based on the proportion of correctly classified studies in the (highest score) ML sample after each iteration. When the proportion of truly relevant studies in the new ML sample is very low, it can be concluded that the proportion of relevant studies that are not being identified by the ML algorithm is also small. Formally, we plan to continue the iterations until statistical testing suggests that we can reject a null hypothesis that <90% of truly relevant studies with a confidence level of 90%. However, the stopping rule will be reviewed and amended as appropriate in the light of the results of processing the initial batches of studies to ensure an efficient and manageable process.

### Data management

Meta-data classifying studies by their basic characteristics will be added to the database through a semi-automated tagging process. Tags will be assigned to all the studies identified through bibliographic databases at the point of downloading their records. Tags will correspond to the specific thematic sub-block of the search strategy through which the study is identified (see column 2 in extended data ( Belesova *et al.*, 2021)) and to the city name detected by the city names filter:

(1) **field of study:** mitigation, adaptation, both;
(2) **health impacts:** general health, nutritional, cardio- and cerebro-vascular, respiratory, renal, thermal effects, other non-communicable diseases, allergies, birth-related, developmental, accidents/injury/violence, vector-borne, helminthic, water-/food-related, other infectious diseases, health behaviours, mental disorders, other, general wellbeing, emotions, cognitive, social psychology.
(3) **sector(-s):** buildings, transport, urban planning and greenspace, food, waste and circular economy, water, energy, health sector, unclear from the abstract;
(4) **city**: city name(-s)

For example, if a study is identified by the mitigation search by the combination of its climate terms, terms for mitigation in the transport sector, and terms for accidents, injury and violence and its abstract or title contains the name of the city "Paris", then it will be assigned the following tag values: field of study – mitigation, health impacts – accidents/injury/violence, sector – transport, city – Paris. If the same study is identified by multiple combinations of search term sub-blocks, then it will be assigned all the relevant tag values (before removing its duplicate records from the database). All tag values assigned through this process will be validated by human reviewers at the abstract screenings stage for their consistency with the content of the study abstracts, titles, and keywords. For a selection of studies, the tags will also be validated against the full text versions of the papers, as described in the following section. 

### Stage IV: validation against full texts

***Validation of decisions on study inclusion***. Those articles whose inclusion could not be agreed and a random sample of the studies whose inclusion was agreed at the abstract review stage will be assessed against the inclusion/exclusion criteria by reviewing their full text versions. These will be reviewed by two independent reviewers, recording reasons for any discrepancies of the decision on inclusion based on full text review *vs* the decision based on abstract review. Any disagreements on inclusion will be discussed and agreed by consensus, involving a third reviewer, if necessary.

***Tag validation***. Accuracy of the meta-data contained in study tags will be verified during the full text review for the same sample of studies whose inclusion is verified at the full text level as well as any studies whose tags could not be previously credibly validated against the information contained in the abstract. The verification will be performed by two reviewers independently and any inconsistencies will be resolved consulting a third reviewer, if necessary.

## Ethics, outputs, and dissemination

The CUSSH project, of which this research is part, has overall ethical approval from the University College London Ethics Committee. Results of the database, including the distribution, variation, and diversity of actions across the world's cities, will be presented in a peer-reviewed academic paper. Furthermore, a series of protocols for systematic reviews that will be produced drawing on the database content and addressing specific research questions, including questions on the success of climate actions and their implementation strategies. Results of the subsequent systematic reviews will be published in peer-reviewed academic journals and additionally communicated to stakeholders through policy briefs and other preferred dissemination formats.

## Study status

The study protocol and search strategy have been completed and searches have been performed; as of publication, screening and subsequent processes have not been started yet.

## Discussion

This protocol described the systematic approach for the development of the first comprehensive database of peer-reviewed studies reporting climate change mitigation and adaptation actions of relevance to human health and wellbeing that have been implemented in cities.

A unique aspect of the database is the innovative use of machine learning methods. All approaches to systematic literature searching have their limitations ( Berrang-Ford *et al.*, 2021) but our deployment of machine learning to enhance the process is designed to make it possible to screen a larger number of studies than is usually feasible through manual review alone and to improve the consistency of the selection process. This is particularly important in our case because urban climate change adaptation and mitigation measures can take many forms and thus require an extensive set of search terms and judgement on the relevance of a disparate array of interventions and study types. While there is a substantial gain in efficiency in terms of the proportion of articles that need to be reviewed manually, a large investment of effort is still needed for the supervised training of the ML algorithm and the repeated checking of results. We are still exploring the most efficient ways to use the ML methods, including the stopping rules for the supervised training and how much further review is undertaken of the studies classified by the fully trained ML algorithm. We will also assess the overall quality of the search using random samples of studies and use the results to guide the refinement of future searches. Without wishing to pre-judge the results, our hope and expectation is that the support of machine learning will allow our review to be more comprehensive than any similar initiative to date.

Our search strategies capture climate change adaptation and mitigation actions, including relevant adaptation and mitigation actions that are not explicitly identified in those terms, as well as studies reporting physical, mental and social health and wellbeing. Mental health and wellbeing have been under-represented in the literature on climate change and health but represent a substantial health burden where they have been assessed ( Berry *et al.*, 2018; Hayes *et al.*, 2018).

We acknowledge that the database is subject to a range of possible sources of bias that are characteristic of systematic reviews:

- **Reviewer bias** can originate from different interpretation of inclusion and exclusion criteria by reviewers, which can also introduce bias into the article classification by the machine learning algorithm. To minimise the risk of such bias we will train all reviewers in consistent application of inclusion and exclusion criteria prior to the start of the machine learning algorithm training. Furthermore, each abstract classification will be performed by two reviewers independently and reconciled with a third reviewer in cases of any disagreement.
- **Publication bias** is likely to be a limitation of the database, as studies on climate change mitigation and adaptation actions in cities tend to be more frequently conducted in high-income countries than low- and middle-income countries, introducing geographical publication bias. The geographical publication bias is likely to be further exacerbated by our language and publication type inclusion criteria. In low- and middle- income countries there is likely to be a higher fraction of grey literature reports on urban climate action and health in local languages than peer-reviewed scientific studies in English. Furthermore, actions that are perceived as successful are more likely to be reported in academic literature than actions that did not have a marked perceived effect. This is likely to introduce another dimension of likely publication bias in our database.
- **Inconsistent definitions** across studies may add further bias, as, for example, study authors may use such terms as ‘climate action’ differently. To minimise bias that can result from inconsistent definitions, we set our own broad definitions for the key terms whose definitions are particularly likely to vary across studies. Any data entry and study classification in the development of our database will be based on these unifying definitions.

The publication bias and strength of the body of evidence will be formally assessed as a part of the subsequent systematic reviews using the content of this database.

The database will have a broad and comprehensive scope with the aim of creating a representative evidence base for the subsequent systematic reviews and flexibility to address new research questions. Only including studies published in English may seem an important limitation. However, we tested our search strategy on studies published in languages other than English (Spanish, Chinese, French, German, Russian, and Arabic) but with an abstract available in English. We found that such studies added only 2% to our overall search results. It is possible that a higher number of studies in other languages would be retrieved if our search strategy was fully translated into these languages. However, in light of resource constraints we took the decision to exclude languages other than English.

We will continue to refine the methods described here as we begin to exploit and learn from the database. Future planned developments of machine learning capabilities include development of searches through further bibliographic databases, including grey literature databases, search engines such as Google Scholar and organisational websites, and consideration of approaches to covering more languages. Together with stakeholders such as C40, we intend to develop a strategy for curation and regular updating of the database and mechanisms for accessing its content. We welcome suggestions for further elaboration and development of the strategy, technical aspects and applications of our database, including suggestions for joint research work.

## Conclusion

This protocol describes an approach for the systematic development of a database of climate change mitigation and adaptation actions relevant to human health and wellbeing. To address the challenge of the broad landscape of published research on such actions across multiple disciplines, sectors, and complex systems, we exploit machine learning methods applied to specialised search strategies to develop a comprehensive and updatable collection of relevant peer-reviewed studies classified by their key characteristics. The database is intended to serve as a source for subsequent systematic reviews addressing specific research questions of relevance to stakeholders in urban climate action and health. The outputs of the systematic reviews will be of value to the scientific community, international networks on city climate action and leadership, such as C40 cities network, urban policy makers, and other stakeholders.

## Data availability

### Underlying data

There is no data associated with this article.

### Extended data

LSHTM Data Compass: Search strategies for: "Climate action for health and wellbeing in cities: a protocol for the systematic development of a database of peer-reviewed studies using machine learning methods". https://doi.org/10.17037/DATA.00002094 ( Belesova *et al.*, 2021)

Data are available under the terms of the Creative Commons Attribution 3.0 Unported (CC-BY 3.0).

## Footnotes

1 We will also include studies with changes in those exposures that are known to have strong links to health and wellbeing outcomes, even if these studies do not measure the outcomes
