# Supplementary material for: Climate action for health and wellbeing in cities: a protocol for the systematic development of a database of peer-reviewed studies using machine learning methods
Source: Wellcome Open Res. 2021 Mar 5;6:50. [Version 1] doi: 10.12688/wellcomeopenres.16570.1 (PMC8022210; doi:10.12688/wellcomeopenres.16570.1)
Supplement: Peer review history

# Peer review: Reviewer response for version 1

**DOI:** 10.21956/wellcomeopenres.18260.r42960

**Published:** 2021-04-01

**Authors:** Tara Neville

**Affiliations:** 1 Environment, Climate Change and Health Department, World Health Organization, Geneva, Switzerland

The authors have provided a thorough, sound and interesting protocol for developing a database of peer-reviewed studies of city-level climate action for health and well-being. The need for this database was clearly articulated, including the critical role of cities in emissions reductions, considerations of the potential benefit or harm of climate action on human health and the current lack of an accessible, comprehensive base of evidence that stakeholders can use to inform impactful action. The protocol, which utilizes a machine learning approach, is innovative and demonstrates an effective solution to the challenge of reviewing a large body of literature that spans numerous sectors and topics. This paper is well organized and the key concepts, methods and processes are clearly presented. The potential biases and/or limitations of this approach have been thoroughly considered. The scope and search strategy are logical and well elaborated. One suggestion for consideration is to include in the search not only population health outcomes but also to include city-level climate action related to health services/health facilities.

 The protocol presented and its application to develop a database on climate action for health and well-being provide a valuable contribution to this field of work and will benefit a wide-range of stakeholders.

 Suggested small revisions/edits:

- p. 3, second paragraph: Clarify the statement on 10,000 climate actions being taken (and further 26,000). Is there an approximate breakdown on whether this is primarily mitigation or adaptation action or scope, in order to provide some context on the next statement of action being taken but little progress in emissions reductions?
- Intro/p.5 literature search – in justifying the need for a machine learning approach over solely human review it might be helpful to expand on the approximate number of the studies that are captured in initial selection/screening steps. (650,000 records is mentioned before filters).
- P 4. PICO statement/Inclusion criteria: consider the inclusion of outcomes related to health services/health facilities and study-types intended to impact vulnerable populations within cities to capture health equity.

Is the study design appropriate for the research question?

Yes

Is the rationale for, and objectives of, the study clearly described?

Yes

Are sufficient details of the methods provided to allow replication by others?

Yes

Are the datasets clearly presented in a useable and accessible format?

Not applicable

Reviewer Expertise:

Monitoring and evidence related to national health sector response to climate change, health benefits of climate mitigation.

I confirm that I have read this submission and believe that I have an appropriate level of expertise to confirm that it is of an acceptable scientific standard.

# Peer review: Reviewer response for version 1

**DOI:** 10.21956/wellcomeopenres.18260.r42963

**Published:** 2021-03-19

**Authors:** Waleska Teixeira Caiaffa

**Affiliations:** 1 Department of Preventive and Social Medicine, Federal University of Minas Gerais Observatory for Urban Health in Belo Horizonte, Belo Horizonte, Brazil

General comments:

 This study protocol aims to develop a systematic record of peer-reviewed academic literature reporting climate and health actions in cities. Justification for this initiative goes from the absence of a comprehensive and innovative approach using a systematic development of a database of studies in cities of climate change to the knowledge of mitigation and adaptation actions and their (dis)benefits for human health and wellbeing. The final aim is to develop a robust knowledge on high-impact climate actions of benefit to human health and informing future action plans, the possible deployment, and scaling-up.

 The authors started the manuscript assembling key concepts comprising elementary terms such as health and well-being to mitigation and adaptation concepts. In the next step, they framed the database's scope using a PICO statement and clear inclusion/exclusion criteria. As an innovative approach, the authors adopted the literature search and screening a combination of machine learning and human processing. As a result, a broad landscape of relevant literature scattered across many disciplines and sectors, usually difficult to be obtained by traditional literature review methods.

 The topic is novel and worthwhile. The analytical approach is fascinating and innovative. The discussion is interesting and timely. However, as a suggestion, some results, even preliminary, as an essential part of the narrative, would add to the manuscript aggregating a lot of value and making it far more interesting to be read.

 Very few revisions/suggestions:

1. On page 8 # 11: Is the section stage IV or stage III? Please, verify.
2. On page 9# 11: I would strongly recommend introducing a new and brief section named preliminary results where the authors could include some information according to the three-stage process of study identification (initial selection, machine learning, and eligibility assessment).

Is the study design appropriate for the research question?

Yes

Is the rationale for, and objectives of, the study clearly described?

Yes

Are sufficient details of the methods provided to allow replication by others?

Yes

Are the datasets clearly presented in a useable and accessible format?

Not applicable

Reviewer Expertise:

Urban health and health inequalities, with a particular focus on social and building urban environments, including urban interventions from the health and non-health sectors.

I confirm that I have read this submission and believe that I have an appropriate level of expertise to confirm that it is of an acceptable scientific standard.
